# Recent Development of MOF-Based Photothermal Agent for Tumor Ablation

**DOI:** 10.3389/fchem.2022.841316

**Published:** 2022-03-16

**Authors:** Xiuzhao Yin, Fujin Ai, Linbo Han

**Affiliations:** ^1^ College of Applied Technology, Shenzhen University, Shenzhen, China; ^2^ College of Health Science and Environmental Engineering, Shenzhen Technology University, Shenzhen, China

**Keywords:** MOFs, photothermal agent, phototherapy, therapeutic modality, ablation

## Abstract

Metal-organic frameworks (MOFs) are 3D-architecture compounds of metal ions and organic molecules with sufficient and permanent porosity, showing great potential as a versatile platform to load various functional moieties to endow the hybrid materials with specific applications. Currently, a variety of photothermal nanometals have been embedded into organic ligands for integrating the unique photothermal effects with the merits of MOFs to improve their performances for cancer therapy. In this review, we have summarized a series of novel MOF-based photothermal materials for this unique therapeutic modality against tumors from three main aspects according to their chemical compositions and structures, i) metal-doped MOF, ii) organic-doped MOF, and iii) polymer-coated MOF. In addition, we have summarized the latest developments and characteristics of MOF-based photothermal agents, such as good biocompatibility, low toxicity, and responsive photothermal conversion without destroying the structure of hybrid photothermal agent. At last, we addressed the future perspectives of MOF-based photothermal agent in the field of phototherapy.

## Introduction

Cancer is a threat that humans need to deal with it urgently; however, effective treatment for cancer therapy is still a challenge (Li et al., 2013a; Probst et al., 2013; Li et al., 2014a; Chen et al., 2016). Currently, chemotherapy, radiotherapy, and surgery are conventional cancer treatments. The side effects of these therapies are not only toxic, but cause panic to patients (Liang et al., 2016; Song et al., 2016; Jia et al., 2021). Therefore, minimally invasive medical technology causes much research interest, which could potentially avoid the above disadvantages. Photothermal therapy (PTT) is an important technique of minimally invasive therapy; in recent decades, near-infrared laser (NIR)-responsive photothermal treatments have become a promising technique for cancer treatment taking advantage of enhanced permeability and retention (EPR) effect (He et al., 2016; Jiang et al., 2021). NIR is a commonly used bio-safe laser source, which shows a decent biological tissue penetration ability and small attenuation. Thus, the NIR laser could reach the photothermal agent (PTA) underneath the skin and transfer the light energy into heat, realizing the increase of local temperature to kill cancer cells. The effectiveness of photothermal therapy has been demonstrated in tumor treatment of mouse model (Chen et al., 1997). In the last few years, various PTAs based on NIR laser have been reported. The key of photothermal therapy is to develop efficient, biocompatible, and targeted photothermal conversion agents (PCAs) (Tang et al., 2018; Mengying et al., 2021).

The conventional PCAs have been deeply developed, such as organic dyes (Kai et al., 2012a), carbon nanomaterials (Zhu et al., 2016), inorganic semiconductor materials (Tian et al., 2011; Li et al., 2013b; Liu et al., 2014a; Song et al., 2015), and noble metal materials (Deng et al., 2016; Wang et al., 2020; Xu et al., 2020). Gold is a common noble metal, and gold nanoparticles with various shapes have been synthesized, such as spherical, rectangular, and hexagonal (Li et al., 2012; Li et al., 2014b; Li et al., 2014c; Jing et al., 2014; Rengan et al., 2015; Jiang et al., 2018; Li and Yin, 2019). The absorption coefficient and photothermal conversion efficiency depend on their morphology, size, and nanostructures of noble metal, while the high price prevents it from being widely used in industrialization. The second type of PCAs comes from carbon-based photothermal materials, such as mesoporous silica, flake graphene oxide, and hollow carbon nanotubes (Chen et al., 2013; Li et al., 2019a; Tsoy et al., 2019; Pereira et al., 2020). Carbon-based light-to-heat PCAs have many advantages, such as relatively high stability, not being easy to be oxidized, low toxicity, and good metabolism in the body. However, their application is limited due to the low photothermal conversion efficiency, and the photothermal mechanism is not clarified. The third type of light-to-heat PCAs is a family of organic dyes, including dyes, polyaniline and polypyrrole nanoparticles, and so on (Chen et al., 1995; Yang et al., 2011; Zha et al., 2013; Chen et al., 2014; Huang et al., 2015; Alves et al., 2018; Li et al., 2020). This kind of material is relatively small in size, which is easy to be swallowed by cells, and its fluorescence can be used for imaging. The disadvantage is that it is difficult to metabolize or maintain in body for a long time, and those dyes are somehow poisonous. The last type of light-to-heat PCAs are inorganic semiconductor materials, such as spherical copper sulfide, flower-like bismuth sulfide, and iron sulfide with a core-shell structure (Hu et al., 2003). The advantage of this type of material is that it has high photothermal conversion with an easy-prepared uniform morphology, and the price of such material is relatively cheap. The disadvantage is that high concentrations of metal ions are toxic and will cause everlasting threat to creatures.

Metal–organic frameworks (MOFs) with porous 3D structures are mostly used as carriers owing to their large surface area, which makes it easy to load drugs (Wang et al., 2015). This special structure composed of organic and inorganic components endows MOFs with various applications (He et al., 2015; Simon-Yarza et al., 2017; Wang et al., 2017), such as gas storage and adsorption (He et al., 2014), functionalized catalyst, and high-efficiency carrier (Bae et al., 2010; Horcajada et al., 2010; Kreno et al., 2010; Chacón-Parra et al., 2021). Some MOFs are inherently fluorescent and can be used for imaging *in vivo*. Various properties of MOFs materials play a unique role in different fields, such as accelerated adsorption, desorption kinetics, and improved bioavailability (Sakata et al., 2013). Some researchers have integrated some properties of biology, physics, and chemistry into MOFs to make them multifunctional and play multiple roles in one field.

According to the current research field of MOFs, this review focuses on the unique functionality of elaborately designed nanoplatforms to generate heat for photothermal therapy of cancer (Zhou et al., 2021; Zhong et al., 2020; Liu et al., 2018) (Table 1). By controlling the size, composition, and other parameters of photothermal agent, as well as its hybrid structure, an MOF-based PTA with optimized photothermal performance is highly desirable (Lan et al., 2019; Hu et al., 2020; Su et al., 2021). Polyethylene glycol (PEG) is widely employed to enhance the hydrophilicity and biocompatibility of MOF-based materials for biomedical applications. This review mainly summarized the combination method, performance test, and application of MOF materials and PTAs for tumor treatment. For example, PTT or photodynamic therapy (PDT) based on MOF-PTA compounds have been developed for this unique therapeutic modality against tumors, including Au NR@ZIF-8, UCNPs@MOF@MIL-100(Fe), Cu-TCPP@MOF, Zr-PDI@MOF, PPy@MOF, and PDA@MOF. The combination of photothermal reagent and MOF materials provides researchers a novel agent for tumor treatment; the photothermal reagents could produce heat under the stimulation of external lasers for thermal ablation of tumors, while MOF materials have the properties of PDT or fluorescence, promoting theranostic effects synergistically for cancer treatment (Table 2). In this review, MOF-conjugate materials as photothermal reagents have been briefly divided into three categories based on their compositions and structures: i) metal-doped MOF, ii) organic-doped MOF, and iii) polymer-coated MOF.

**TABLE 1:** Summary of MOFs used as photothermal theranostic platforms

MOFs	MOF skeleton components	Therapeutic option	Animal model	References
AU@MOF-DOX	Zn^2+^, 2-H-MeIM	Chemotherapy, PTT	H22 Tumor-bearing mice	(Sun et al., 2012; Chen et al., 2017a; Lin et al., 2017; Koschnick et al., 2021; Li et al., 2021)
MGH	Fe^3+^, BTC	CDT, PT, starvation therapy	4T1 Tumor-bearing mice	(Liu et al., 2014b; Lu et al., 2015; Ibacache et al., 2016; Yang et al., 2018)
HUC- PEG	Hf^4+^, BDC, TCPC	PTT, PDT	U14 Tumor-bearing mice	(Sun et al., 2012; Zhang et al., 2013; Wang et al., 2016)
LA-AUNR/ZIF-8	Zn^2+^, 2-H-MeIM	Chemotherapy, PTT	H22 Tumor-bearing mice	(Hu et al., 2009; Tian et al., 2017; Deng et al., 2019)
B9-MIL@cat-fML	Fe^3+^, NH2-BDC	PTT, PDT	HeLa Tumor-bearing mice	(Lee et al., 2012; Yang et al., 2012; Ercius et al., 2015; Cheon et al., 2016; Falcaro et al., 2016)
Cu-TCPP MOF	4-carboxyphenyl, porphyrin	PTT, PDT	Saos-2 Tumor-bearing mice	(Li et al., 2014d; Alexander Ardagh et al., 2016; Li et al., 2019b; Han et al., 2020)
Cu@MOF	PCN 224	Chemotherapy, PTT	NIH3T3 Tumor-bearing mice	(Lee et al., 1999; Struijk et al., 2000; Chen et al., 2015; Würthner et al., 2016)
siRNA/Zr- FeP MOF	Zr-FeP	PTT, PDT	MCF-7Tumor-bearing mice	(Mahara et al., 2002; Che et al., 2007; Kai et al., 2012b; Park et al., 2012; Ghosh et al., 2014; Tan et al., 2014; Meng et al., 2016; Zeng et al., 2016; Espín et al., 2018; Lü et al., 2019)
PPY@MOF	MTT	PTT	4T1 Tumor-bearing mice	(Liu et al., 2006; Cairns et al., 2016; Seoane et al., 2016; Chen et al., 2017b; Xuechao et al., 2019)
PDA@MOF	Zn^2+^, 2-H-MeIM	Chemotherapy, PTT	4T1 Tumor-bearing mice	(Park et al., 2014; Choi et al., 2015; Liu et al., 2016b)

**TABLE 2:** The advantages and disadvantages of the three categories

MOFs	Advantages	Disadvantages
Metal-doped MOF	PTA/MOF materials with designed functionalities, such as fluorescence imaging, chemo-photothermal therapy and controlled drug release	Low yield of nanoparticles, low biocompatibility
Organic-doped MOF	Low toxicity, magnetic resonance (MR) imaging capability, PDT therapy, controlled drug release, superior photothermal conversion efficiency	Connection between MOF structure and treatment efficiency, chronic toxicity assessment caused by acute toxicity and molecular level
Polymer-coated MOF	Good stability, biocompatibility and degradation performance, stimulus-response multifunctional abilities, chemo-photothermal therapy	Low yield of nanoparticles, redirection of drug release

## Metal-Doped MOF for PTT

Metal-doped MOF refers to the structure formed by metal nanoparticles doped on MOF framework. PTAs with core-shell nanostructures have been combined and applied in the field of tumor treatment due to their good biocompatibility, drug delivery performance, and synergistic effect between core-shell components (Chen and Yin, 2014; Elsabahy et al., 2015). The MOF material has a large specific surface area, and there are many covalent bonds and coordination bonds on it. Before the metal surface being coordinated with MOF, it must undergo a preliminary hydrophilic treatment to connect the metal surface with more affinities, promoting the two to be combined. Once such PTAs loaded on MOF materials, the surface of these hybrids can be easily modified by various functional moieties, endowing the PTA/MOF materials with designed functionalities, such as fluorescence imaging, chemo-photothermal therapy, and controlled drug release.

### Au NPs@ZIF-8 for PTT

Au NPs is widely used as PTAs for photothermal therapy due to its low toxicity and good photothermal convertibility. However, gold nanoparticles with this structure have many shortcomings in the field of tumor treatment, such as poor biodegradability and surface modification. Besides, Au NPs is easy to aggregate *in vivo*. In order to solve these shortcomings of Au NPs, it is highly desirable to develop new types of Au NPs-based functional materials for highly efficient cancer therapy. Au NPs based MOF materials are recently developed as promising hybrid materials to avoid the abovementioned deficiencies.

**FIGURE 1:**
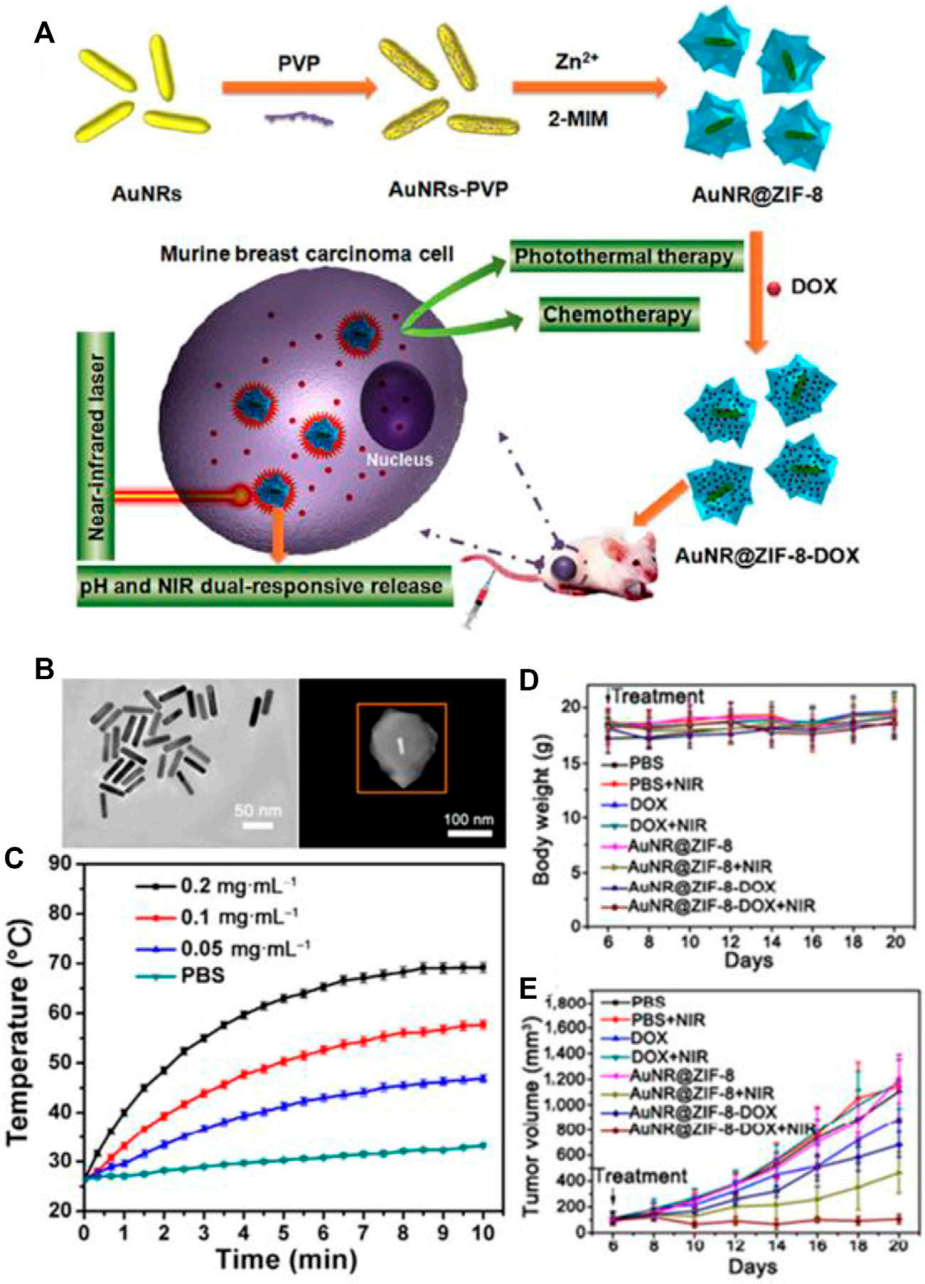
**(A)** Schematic representation of the Au NRs@ZIF-8 core–shell nanostructures. **(B)** TEM image of CTAB-stabilized Au NRs and HAADF-STEM image of single Au NRs@ZIF-8 core shell nanostructure. **(C)** The temperature increased by Au NRs@ZIF-8 core–shell nanostructures in PBS solution l. **(D,E)** Observation of changes in **(D)** body weight and **(E)** relative tumor volume from 4T_1_ tumor-bearing mice with different treatments. Reproduced with permission (Li et al., 2018). Copyright 2018, Nano Research.

Recently, Tang, and co-workers reported a multifunctional nanoplatform-based Au NPs@ZIF-8 for improved multifunctional tumor therapy under NIR irradiation (Li et al., 2018). They have designed the novel Au NRs by the seed-mediated method and then utilized cetyltrimethylammonium bromide (CTAB) to modify the Au NRs surfaces to achieve a better stability (Figure 1A). Finally, they added 2-methylimidazole (2-MIM) to the PVP-stabilized Au NRs for changing surface structure. There are representative Au NPs and typical Au NRs@ZIF-8 with core-shell structure (Figure 1B). The combination of gold nanoparticles and MOFs in PBS solution can heat up to 54°C in 5 min (Figure 1C). However, the PBS of the control group only increased by 2°C under the same power of light. These data indirectly prove that the combination of MOFs and gold nanoparticles also has a strong photothermal effect under the 808-nm NIR laser. The temperature (photo-induced hyperthermia) can be tuned by changing the concentration of the Au NRs@ZIF-8 core–shell nanostructures. For the animal experiments, the safety must be considered, and the weight of mice can reflect the toxicity of PTA-MOF. There was no significant change in body weight within 14 days, indicating that Au NRs@ZIF-8 core-shell nanostructures have lower systemic toxicity (Figure 1D). Mice injected with Au NRs@ZIF-8-DOX complex had the best tumor suppressor effect under near-infrared irradiation, about 90%. In contrast, Au NRs@ZIF-8 core-shell nanostructures injected with near-infrared irradiation (58%) and Au NRs@ZIF-8-DOX complex (30%) without near-infrared irradiation reveal the obvious and effective synergistic effect of photothermal therapy and chemotherapy in the body.

On the other hand, crystalline zeolitic imidazolate framework-8 (ZIF-8) is a classic MOF, which is connected by the coordination between low-toxic Zn^2+^ and 2-MIM. It has a cross three-dimensional structure with guest-matching pore size and large specific surface area, which can significantly increase the drug-carrying capacity and facilitate surface modification in order to accurately deliver drugs or make cells penetrate deeply (Liu and Tang, 2012; Sun et al., 2012). In addition, a series of multifunctional core-shell NPs@ZIF-8 nanostructures, including polyacrylic acid@ZIF-8, CuS@ZIF-8, and graphene quantum dot@ZIF-8 ^62–64^, have been widely used as advanced therapeutic functional nanomaterials.

The Au NRs@ZIF-8 core–shell nanostructures had pH and NIR dual stimuli-responsive drug release. The acidic environment of tumor sites and the local NIR photoconversion heat are greatly beneficial to trigger the break of the coordination bond between DOX and ZIF-8 shell or between Zn^2+^ and the imidazoline of ZIF-8 shell itself, thereby realizing the stimuli-responsive drug release. The multi-modal system based on Au NRs@ZIF-8-DOX complex has good biodegradability.

**FIGURE 2:**
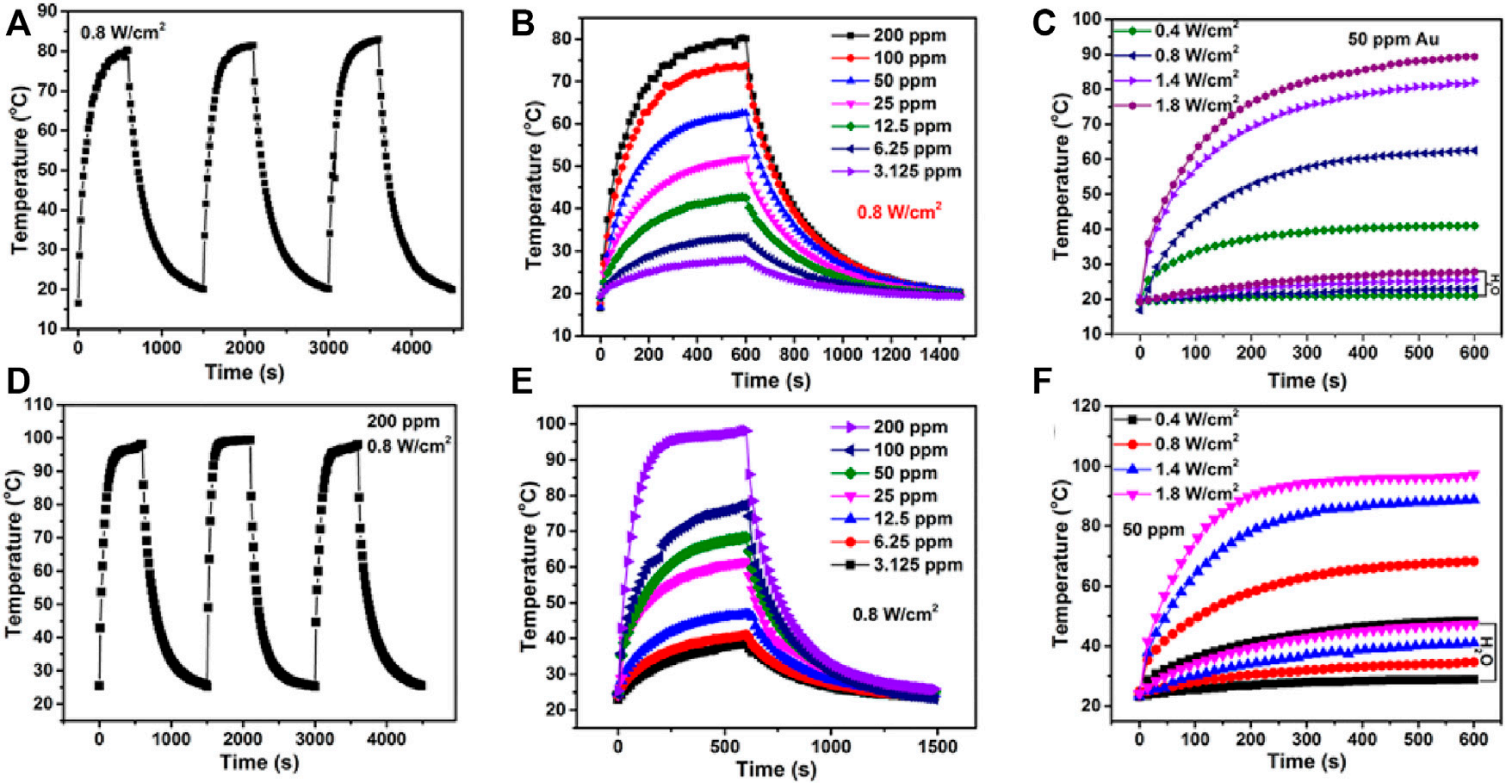
**(A)** Heating and cooling circles for Au@MOF. **(B)** The photothermal performance of the Au@MOF with different concentrations of Au irradiated. **(C)** The temperature increasing and cooling curves of Au@MOF aqueous solution with concentration fixed on 50 ppm Au. **(D–F)** are measured under the 1,064 nm laser irradiation, **(A–C)** are measured under the 808 nm laser irradiation. Reproduced with permission (Deng et al., 2019). Copyright 2019, Nano Letters.

Li and co-workers designed a novel core−shell Au@MOF nanocarrier with NIR-II (Deng et al., 2019). Under the same laser power conditions, NIR-II has a higher penetration depth on the surface of organisms than NIR-I, which is more conducive to the application of photothermal materials in tumor treatment. The temperature (photo-induced hyperthermia) can be tuned by changing the concentration of the Au NRs (Figure 2E). By contrast with H_2_O, the temperature of Au@MOF solution increases to almost 100°C under the same power and the concentration of Au NPs (Figure 2F). These results demonstrated that the Au@MOF was an attractive photothermal agent, and the photothermal conversion efficiency of Au@MOF was 48.5% under 1,064 nm laser, which was higher than that of the representative materials such as Cu_2−x_ S (30.8%), Au nanoplate, and Nb_2_C (46.65%) (Hu et al., 2009; Lin et al., 2017; Li et al., 2021). The combination of gold nanoparticles and MOFs has many advantages: Au NPs will convert light energy into heat energy under the irradiation of NIR. A certain amount of heat will make the cavities on the surface of MOFs larger, and the release rate of drugs caused by heat will also be increased. The more specific surface area of MOFs provides more attachment surfaces for gold particles, so that there are more gold particles per unit area, the light-to-heat conversion efficiency is greatly improved, and the release of drugs is also greatly accelerated.

Compared with NIR-I, the shell Au@MOF shows higher light-to-heat conversion efficiency under 1,064-nm laser irradiation. In addition, in other reports, a new core-shell Au@MOF nanocarrier was successfully prepared, which has high anti-cancer drug delivery ability, pH-sensitive drug release ability, superior NIR-II responsive photothermal conversion ability, and good biocompatibility. In addition, chemotherapy-photothermal combined therapy has achieved significant synergistic effects in inhibiting tumor growth. At the same time, infrared/PAI imaging also shows its superiority in imaging-guided monitoring and treatment.

### UCNPs@MOF@MIL-100(Fe) NPs for PTT

Some research teams have used MOFs materials in the field of cancer treatment (Koschnick et al., 2021). Multifunctional MOFs with iron elements have been used in the treatment of cervical cancer, and MOFs ware regarded as deliver for hydrophobic drug DHA (Chen et al., 2017a). Due to the special structure of MOFs, some of its own components can produce ROS which can poison cancer cells under the irradiation of laser, achieving the effect of photodynamic therapy (PDT) (Liu et al., 2016a; Lu et al., 2016). In addition, MOFs have a large specific surface area and can carry some photothermal reagents. Therefore, it demonstrated further study for cancer theranostics with MOFs, and other potential properties of MOFs materials are slowly being explored by some researchers for clinical translation (Lu et al., 2015).

Interestingly, researchers have developed iron-composite MOFs materials for PTT, PDT, and chemotherapy as a multifunctional treatment method. Due to the 3D structure of MOFs, iron ions can be combined by coordination bonds or covalent bonds. In response to excessive hydrogen peroxide (TME), the element generates a large amount of ROS to achieve the therapeutic effect of PDT. The chemotherapeutic drug DOX can respond under acidic conditions and release the drug in a specific area of the tumor to achieve the purpose of exquisite treatment. The heat generated by PTT can promote the release of drugs and can also kill tumor cells at high temperature. This multi-functional drug delivery platform realizes the combination of multiple treatment methods, which is better than a single treatment effect, and provides a lot of meaningful reference for future clinical transformation.

**FIGURE 3:**
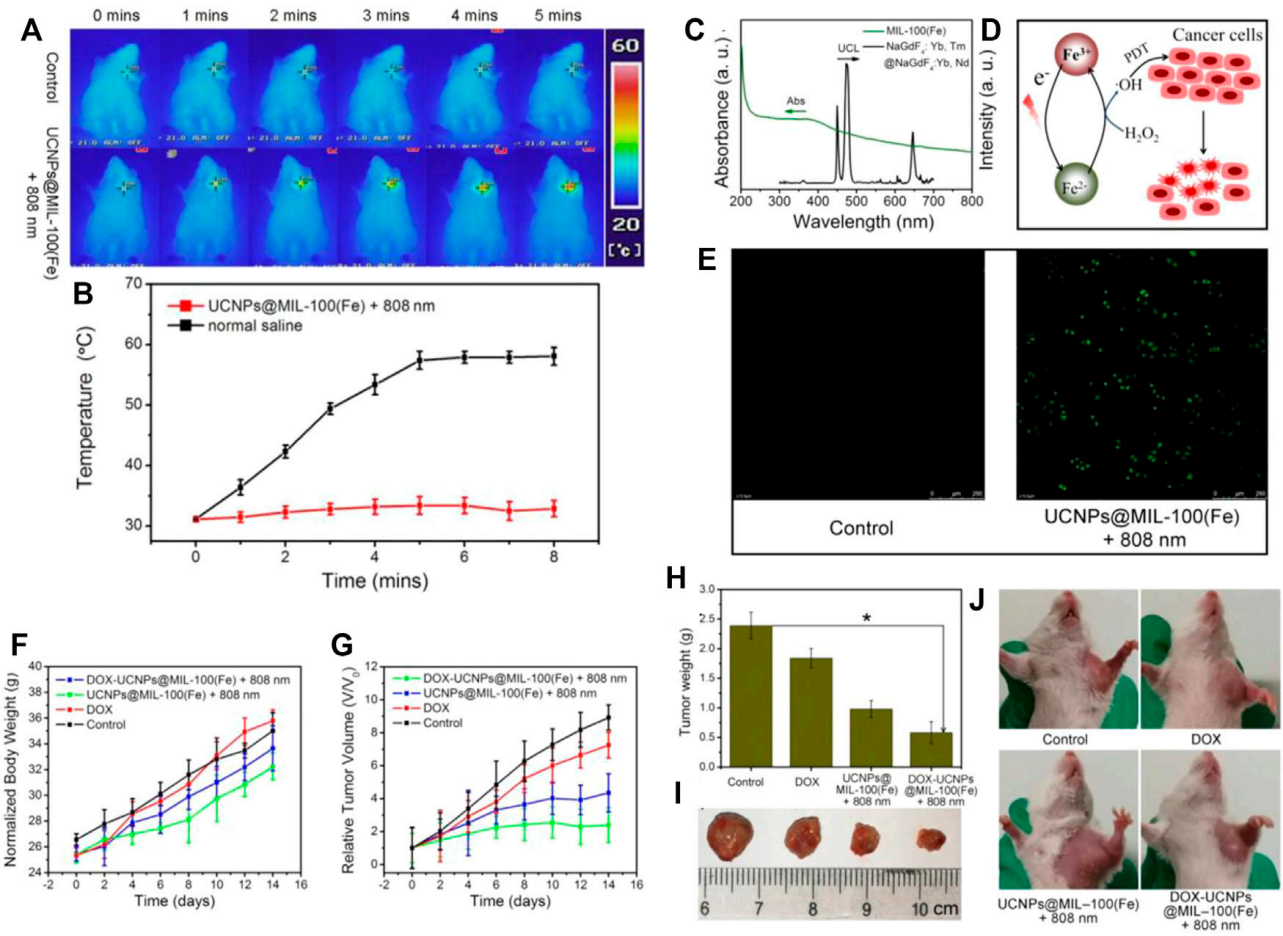
**(A)** Photothermal imaging in mice and **(B)** The temperature rise before and after injection of MOF-Fe nanomaterials. **(C)** The emission spectrum of NaGdF4:Yb,Tm@NaGdF4:Yb and shell of iron ions. **(D)** Schematic diagram of the role of iron ions. **(E)** Confocal image of HeLa cells with different treatment (UCNPs@MIL-100[Fe] NPs and control). **(F)** The weight of mice are treated differently. **(G)** The changes in tumor volume are treated differently. **(H)** Photos of the changes in the body weight and **(I)** tumor volume of mice. **(J)** Physical photos of mice after different treatments with control, pure DOX, and UCNPs@MIL-100(Fe) NPs. Reproduced with permission (Yang et al., 2018) Copyright 2018, Chemical Engineering Journal.

Yang and his team prepared a combination of MOFs and upconversion particles for tumor treatment (Yang et al., 2018) and confirmed that the MIL-100(Fe) shell effectively converts light energy into heat energy for tumor ablation. They also demonstrated that the temperature quickly increased to about 57°C (Figures 3A,B). In addition, the sample produces a large amount of hydroxyl radicals (OH) reactive oxygen species (ROS) in the presence of H_2_O_2_ produced by Fenton reaction, which is highly toxic to tumor cells. In this process, due to the generation of carriers in Fe-O clusters, electrons are transferred from O^2−^ to Fe^3+^ after light stimulation to form reduced Fe^2+^ ions (Figure 3D). Based on the Fenton reaction, the transmitted photons can further react with H_2_O_2_. Free radicals attack cancer cells through the process of photodynamic therapy. The biocompatibility of this nanomaterial is relatively good, and it can be noted that the multi-mode treatment is better than the single-mode treatment (Figure 3G).

Metal-doped MOF have two main advantages, which can adjust the size of the metal particles and the structure of the organic frame depending on elaborate design. On the other hand, this MOF material is capable of loading due to its high specific surface area; it can deliver some drugs, which can be applied to imaging and therapeutics in biomedical sciences.

## Organic-Doped MOF for PPT

Organic inorganic hybrid@MOF is composed by metal compounds and MOFs, whose connection is mainly contributed by polymers. In MOF NPs, organic ligands provide a series of space framework of MOF; inorganic compounds offer metal ions in the reaction. They hybridize each other under certain conditions, forming polycrystalline nanoparticles in the form of covalent bond or coordination bond. What’s more, the polycrystalline nanoparticles have many unique properties, playing an important role in the field of photothermal treatment.

### Ultrathin Cu-TCPP MOF Nanosheets for PTT

Some groups reported that two-dimensional nanosheet photothermal materials have a better photothermal conversion rate than solid photothermal materials due to a larger specific surface area and faster conversion rate (Liu et al., 2014b; Ibacache et al., 2016). Various nanosheets, such as graphene oxide, black phosphorus (Yang et al., 2012; Cheon et al., 2016) Germanium, boron, metal oxides, and transition metal sulfides (Lee et al., 2012), have been demonstrated to have light-heat transforming effects. Recently, some groups have successfully prepared a new member of the 2D MOF materials (Ercius et al., 2015). In particular, these MOFs have specific functionalities by changing the categories of metal ions and ligands (Falcaro et al., 2016), and some reported copper-based nanostructures exhibited NIR light absorption properties (Li et al., 2015), which could effectively generate heating under the 808 nm laser irradiation. In addition, 2D Cu-TCPP MOF nanosheets possess the ability for both SO generation and NIR absorption for phototherapy of cancers. In addition, when the copper nanosheets are not combined with the MOFs, some hydrophilic chemicals will be grafted on copper surface, and those hydrophilic chemical bonds will hybridize with the hydroxyl or carboxyl groups on the surface of the MOFs to form a stable composite, which could be used for cancer treatment.

**FIGURE 4:**
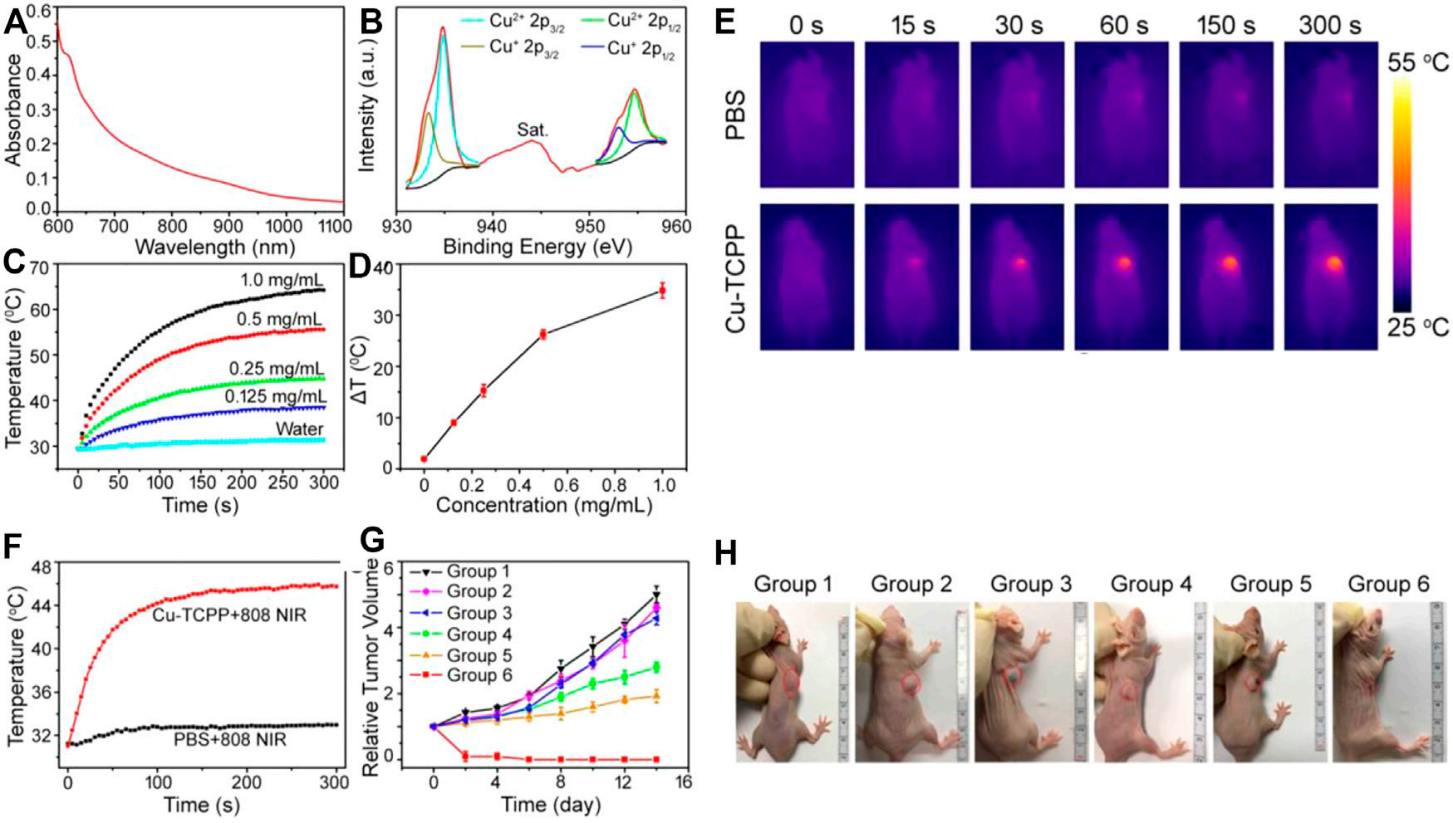
**(A)** UV absorption curve of copper nanosheet MOF. **(B)** Cu 2p XPS spectrum for copper nanosheet MOF. **(C)** Heating curve of different concentrations of copper nanosheet MOF. **(D)** Temperature change curve of different concentrations of MOF material. **(E)** The light-heat curve of mice with light time. **(F)** Change of heating curve before and after adding MOFs. **(G)** Changes in tumor volume in mice after receiving different treatments. **(H)** Picture of changes in mouse tumor volume after different treatments. 1: PBS; 2: Cu nanosheet; 3: PBS + Laser; 4: Cu nanosheet + PDT; 5: Cu nanosheet + PTT; 6: Cu nanosheet + Laser. Reproduced with permission (Li et al., 2019b). Copyright 2018, Theranostics.

Recently, Wu, and co-workers reported a Copper nanosheet MOF material is used for tumor treatment under laser irradiation (Li et al., 2019b) and then demonstrated that photothermal conversion rate of nanosheets was higher than that of grapheme oxide. The previously reported copper-based nanostructures have PTT activity due to non-equivalent ions (Cu^+^ and Cu^2+^), which leads to ionized free carriers to achieve NIR absorption (Figure 4A). According to the valence state of Cu in Cu nanosheet, the binding energy of 944.0 eV is assigned to the oscillating satellite peak of Cu^2+^ (Figure 4B); the Cu 2p 3/2 (934.8 eV) peak and Cu 2p 1/2 (954.7) in the Cu 2p spectrum eV) peak and two oscillating satellites confirmed the coexistence of two copper valence states (Cu^2+^ and Cu^+^) (Alexander Ardagh et al., 2016). The relative atomic percentages of Cu^2+^ and Cu^+^ measured by XPS method were 63% and 37% respectively. This phenomenon is based on the abnormal defect structure and ultra-thin characteristics of Cu-MOF nanosheets, and it is believed that it has a broad spectrum and strong light absorption intensity, and it has abundant copper vacancies. Cu-MOF nanosheets can effectively convert laser energy into heat energy because of strong light absorption at this wavelength (Figures 4C,D). For mice injected with PBS, the surface temperature of the irradiated area increased by less than 2°C under 808 nm laser irradiation. Under the same power, the tumor surface temperature of Cu-MOF nanosheets increased from 31°C to 45°C (Figures 4E,F). The tumor volume of mice reflected therapeutic effect by changing laser irradiation or Cu-TCPP MOF nanosheets injection. Comparing with the other groups (1–3), among them, PDT alone (Group 4) or PTT alone (Group 5) had a slight inhibitory effect on tumor growth. After PTT + PDT combined treatment, the tumors in the six groups completely resolved (Figures 4G,H). They proved that the multi-modal treatment of copper-tcpp MOF nanosheets is an efficient and feasible strategy for phototherapy of cancer cells.

**FIGURE 5:**
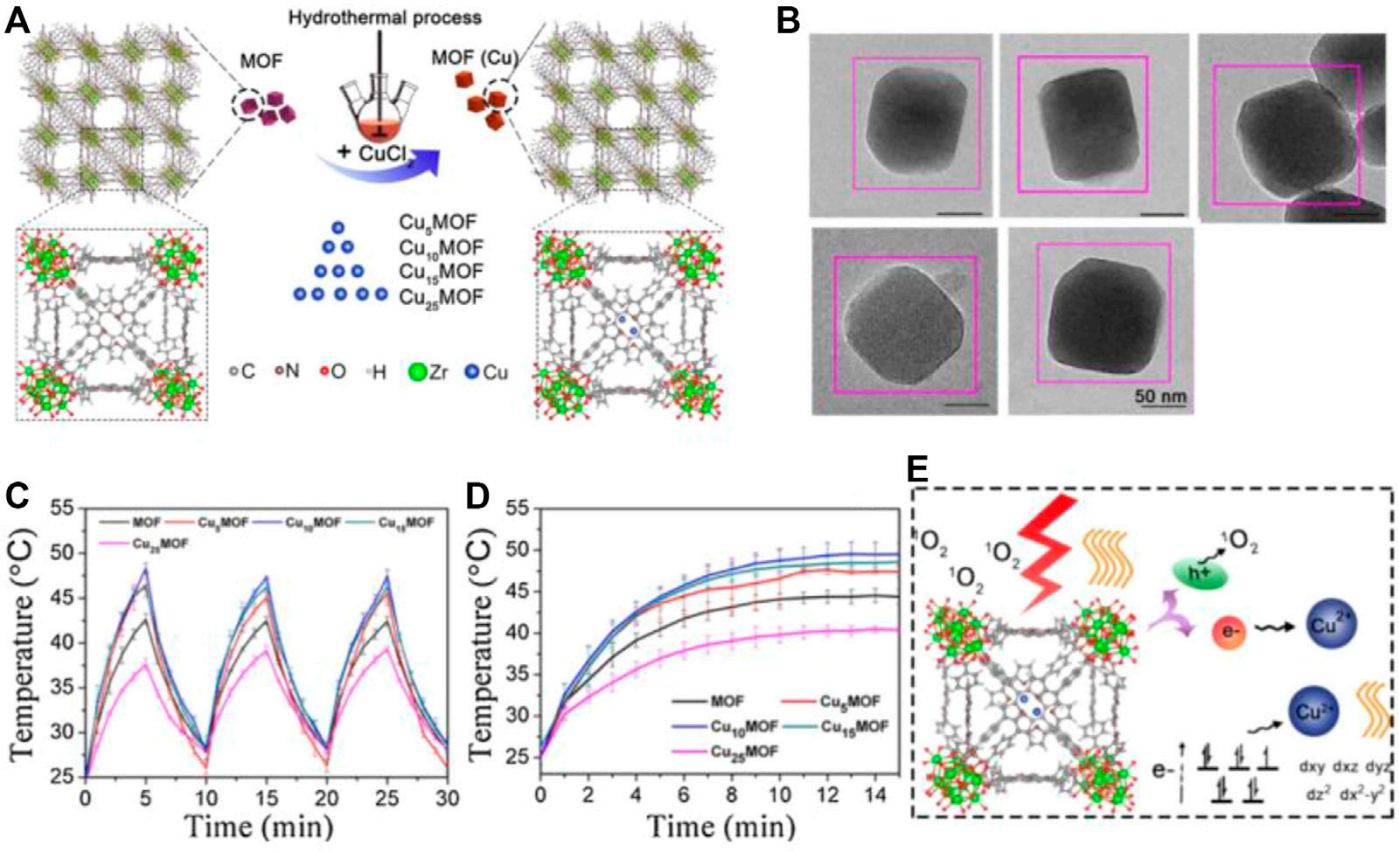
**(A)** Schematic diagram of the preparation of copper MOF material. **(B)** TEM images. **(C)** Heating and cooling curves of copper MOF materials under light. **(D)** Light and heat curves of MOF materials with different copper content, Cu_5_MOF, Cu_10_MOF, and Cu_15_MOF, Cu_25_MOF. **(E)** Schematic diagram of the mechanism of copper MOF material improving photocatalysis and photothermal effect. Reproduced with permission (Han et al., 2020). Copyright 2020, Applied Catalysis B: Environmental.

Very recently, Yuan, and co-workers reported that copper ions and Copper ions and Purin 224 are combined for use in the field of photothermal catalysis 224 are combined for use in the field of photothermal catalysis (Han et al., 2020) (Figure 5A). They demonstrated that the photothermal of Cu_10_MOF had the highest photothermal conversion efficiency and have proven that the MOFs containing porphyrin also showed photothermal properties under 660 nm light (Li et al., 2014d; Luo et al., 2017). The incorporated Cu^2+^ could translate the light energy into heat due to the d-d transition (Figure 5C), and this hybrid can improve the photothermal property of porphyrin by introducing a proper amount of Cu^2+^ into the porphyrin. However, the photothermal conversion of porphyrin will decrease when too much Cu^2+^ is introduced. When the light is over, the temperature of the MOF composite material is lowered to room temperature, and the temperature of the material starts to rise again after the light is taken off again. This phenomenon proves that this MOF material has a good photothermal effect (Figure 5E). The synthesized MOF nanoparticles reacted with CuCl_2_ through a simple hydrothermal method to introduce Cu^2+^ into the porphyrin ring through the formation of NeCu bond, and the series of Cu-doped MOF were termed as Cu_n_MOF (where “n” represented the different molar ratio between Cu and Zr when the Cu was added, indicating Cu was n% of Zr). The structure of the Cu^2+^-doped MOF consisted of Zr_6_ clusters linked with both TCPP and CuTCPP.

In addition, they also demonstrated that Cu_10_MOF didn’t have any appreciable toxicity (Würthner et al., 2016); when skin wounds receive bacterial infection, this MOF material can be used for wound healing and photothermal sterilization. What’s more, the Cu-TCPP MOF nanosheets also possessed T1-weighted magnetic resonance (MR) imaging capability due to the unpaired 3d electrons of copper and also demonstrated that the ultrathin Cu-TCPP MOF nanosheets exhibited ability to produce singlet oxygen because of the inherent characteristic of TCPP. It was noted that Cu_5_MOF was a typical sample with the lowest electron-hole recombination speed; and with an increase in the amount of doped Cu^2+^, the recombination speed of electrons and holes gradually became slightly faster. This occurrence might be because when more Cu^2+^ was introduced, more photo-generated electrons would be trapped, and the reduced metallic Cu would in turn consume the photogenerated holes, resulting in a faster recombination of the electron-hole pairs, especially for Cu_25_MOF. In addition, doping with a proper amount of Cu^2+^ could also reduce the electrical impedance, thereby favoring charge transfer. Herein, designing multifunctional MOF nanostructure as PCAs is a meaning direction for enhancing the multi-theranostic tumor therapy.

### Zr@PDI for Boosting NIR Photothermal Conversion

**FIGURE 6:**
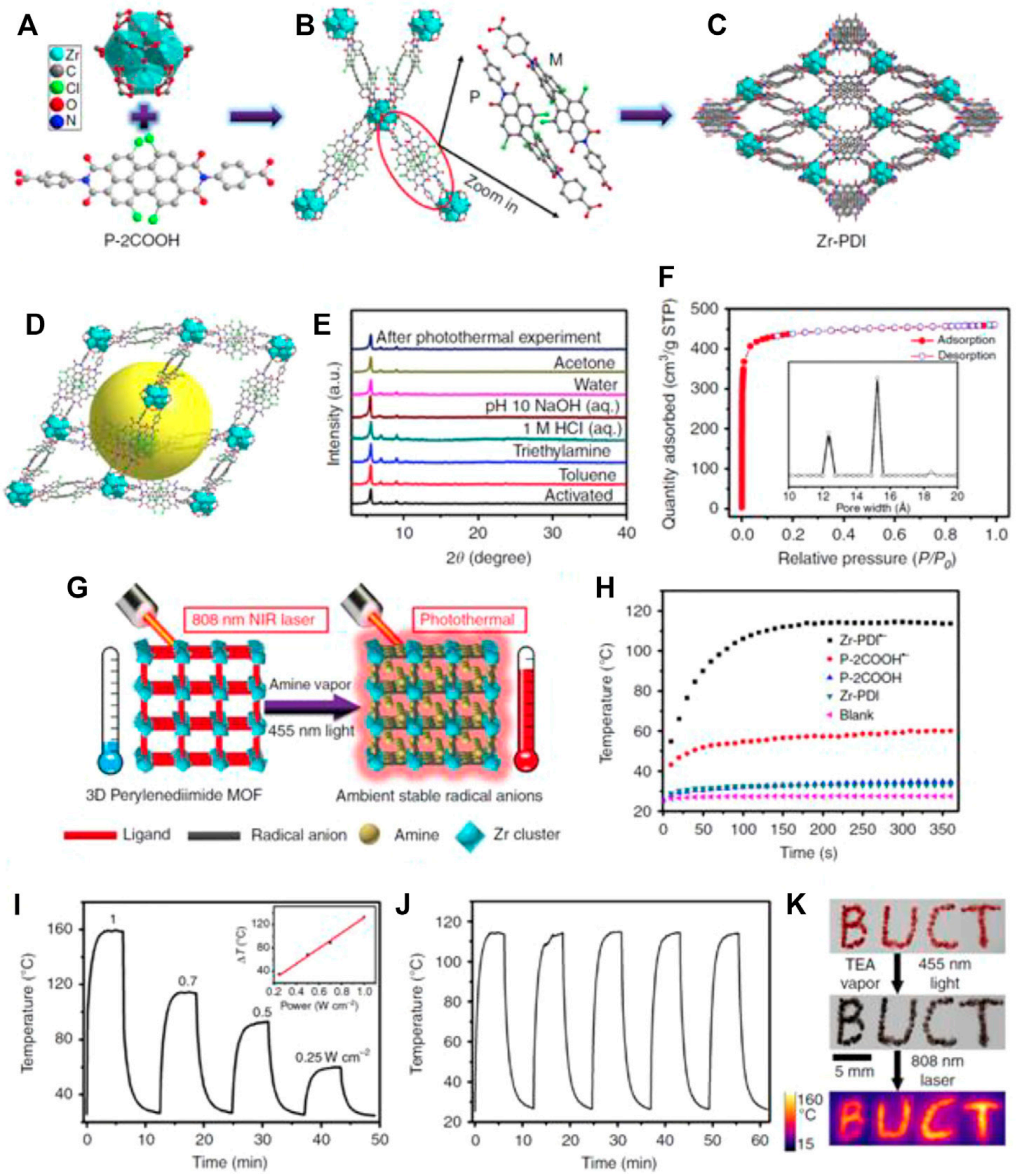
Synthetic schematic diagram of Zr-PDI. **(A)** Schematic diagram of the special structure of Zr and P-2COOH. **(B)** The connection method of Zr and P-2COOH. **(C)** a-Axis crystal structure of Zr-PDI. **(D)** The microstructure of Zr-PDI. **(E)** Light and heat stability curve of Zr-PDI. **(F)** Nitrogen adsorption curve of Zr-PD and pore size distribution. **(G)** Schematic diagram of photothermal conversion of Zr-PD. **(H)** Photothermal conversion curves of Zr-PDI^•−^ film on quartz glass. **(I)** Heating and cooling curves of Zr-PD at different optical powers. **(J)** Light and heat stability curve of Zr-PDI. **(K)** Photothermal digital picture of Zr-PDI-BUCT. Reproduced with permission (Lü et al., 2019). Copyright 2019, Nature Communication.

Perylenediimides (PDIs) are an organic dye that can be used in the preparation of biological materials (Struijk et al., 2000; Chen et al., 2015). PDI can be converted into freely movable anions (RAs) under the change of external conditions. These anions can be used for photocatalysis and cancer treatment (Lee et al., 1999; Che et al., 2007; Kai et al., 2012b; Ghosh et al., 2014). Under the stimulation of near-infrared light, RAs can generate heat for the thermal ablation of tumors (Park et al., 2012; Tan et al., 2014; Meng et al., 2016). Recently, Yin and co-workers reported a Zr-PDI^•−^ as a NIR photothermal material (Lü et al., 2019), which demonstrated higher light and thermal stability and high specific surface area. Zr-PDI forms a fascinating 3D grid, which is regarded as sheet to capture electron donors, so as to achieve the effect of photoelectron conversion (PET) (Figures 6A–D).

In comparison, the temperature of the quartz glass coated with Zr-PDI rose to 114°C under NIR laser irradiation, while blank quartz glass increased by only 2.4°C (Figure 6H). They demonstrated that Zr-PDI^•−^ had good photothermal conversion efficiency (52.3%), which was much higher than latest reported traditional materials such as Au nanorods (21.0%), organic cocrystals (18.8%), and selenophene-derived polymer films (40%) (Espín et al., 2018; Zeng et al., 2016; Mahara et al., 2002; Kim et al., 2013). The photothermal effect is linearly dependent on the NIR laser power from 0.25 to 1W cm^−2^, an indication of a thermal control performance (Figure 6I). They also demonstrated the high stability of Zr-PDI^•−^ at high temperatures, which was attributed to the effective NIR absorbance of TEA, TPA, EDA-loaded Zr-PDI^•−^, and displayed the potential of the RAs in photothermal imaging (Figure 6K). The high temperature of Zr-PDI^•−^ has great potential for bio-imaging and biomedical applications such as photothermal therapy (Zheng et al., 2015; Chen et al., 2017b; Zhang et al., 2017). The combination of Zr nanoparticles and MOFs has many advantages: a PDI-based 3D MOF (Zr-PDI) with ultrastable RAs provides a unique platform for NIR photothermal conversion. The suitable pores allow electron donor amine vapors to occupy the cages of Zr-PDI. Upon irradiation with blue light, black Zr-PDI^•−^ with NIR absorbance can be formed through PET. The produced RAs, which are in the Zr-PDI cages, can stay unobstructed under ambient conditions for at least a month. So, a strategy to stabilize PDI^•−^ without complicated design and tedious synthesis was discovered. Under 808 nm laser irradiation, the temperature of the Zr-PDI^•−^ sharply increases; it has a superior photothermal conversion efficiency due to non-radiative pathway. With post-synthesis modifications, this MOF material of outstanding stability has great potential in biomedical applications such as bio-imaging and photothermal therapy.

## Polymer-Coated MOF for PTT

Polymer-coated MOF refers to a MOF material that has been coated by photothermal polymers. The high specific surface area of MOF provides coatings with more footholds, so that the common advantages of the two can be brought into full play*.* Polydopamine (PDA) and polypyrrole (PPy), as versatile coating material in surface treatment, combined with MOF, emerged as excellent photothermal transduction, because these coating materials have strong and wide near-infrared absorption. Comparing with other photomal reagents, PPy has good stability, biocompatibility, and degradation performance. These properties can be applied in the biomedical field perfectly. On the other hand, some groups demonstrated that the combination of polydopamine and MOFs, a multifunctional coating material, makes it easy to integrate different functional therapies to obtain stimulus-response multifunctional MOFs, with a wide range of photothermal efficiency and outstanding ability to eliminate tumors through chemo-photothermal therapy.

### Polypyrrole-Coated MOF for PTT

**FIGURE 7:**
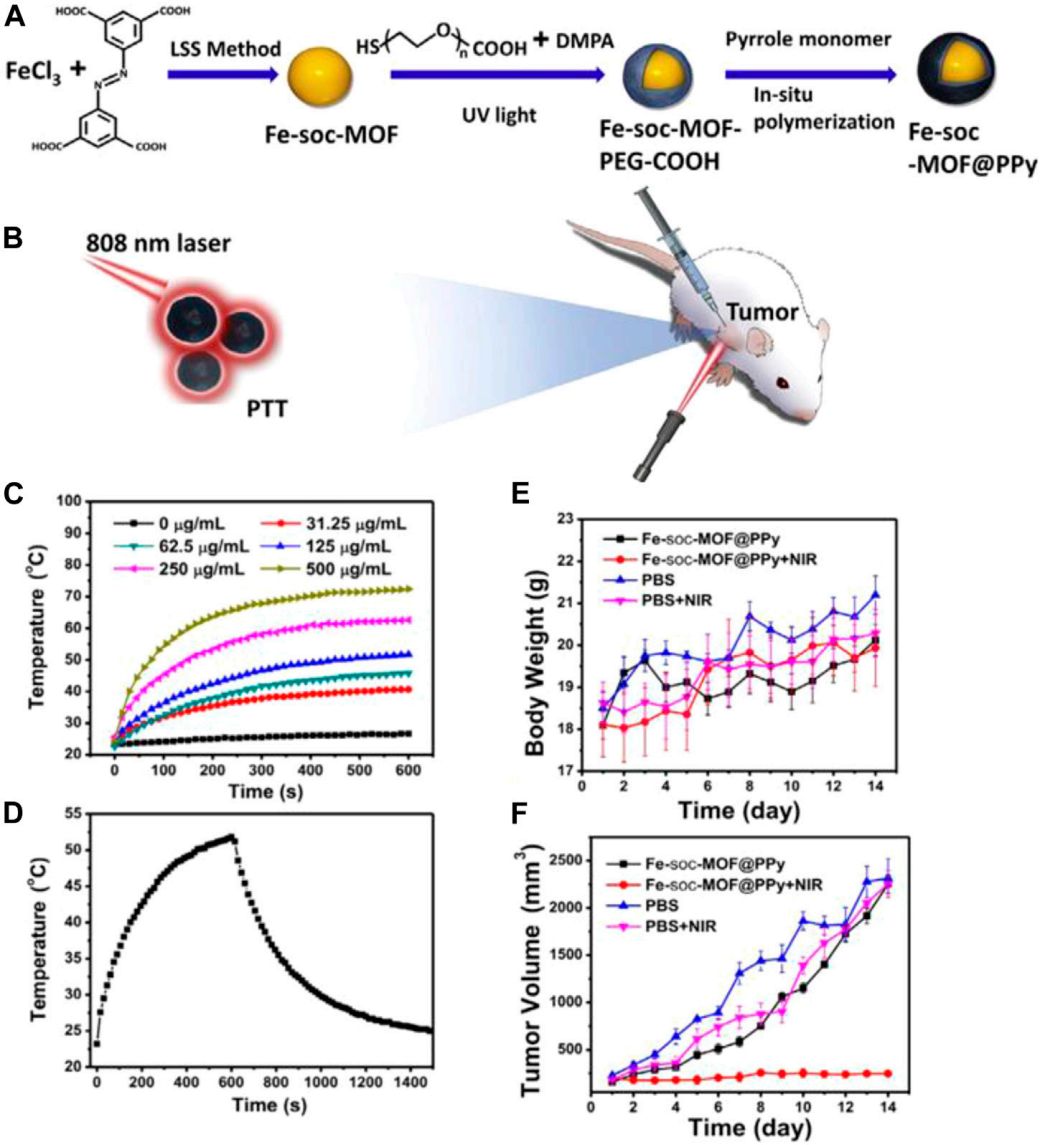
**(A)** Schematic diagram of Fe-soc-MOF@PPy composite material synthesis; **(B)** 808 nm Laser for PTT. **(C)** Different concentration of Fe-soc-MOF@PPy heating curve. **(D)** Fe-soc-MOF@PPy heating and cooling curve. **(E)** The body weight change curve of mice after different treatments. **(F)** Tumor volume change curve after different treatments in mice, respectively. Reproduced with permission (Xuechao et al., 2019). Copyright 2019, Chemical Engineering Journal.

The combination of iron MOF composite materials for tumor treatment has been reported with the linker of azobenzenetetracarboxylicacid (H4-ABTC) ligand (Liu et al., 2006; Cairns et al., 2016). Recently, Lin and co-workers reported a PPy-coated Fe-soc-MOF as multifunctional theranostic platform (Seoane et al., 2016; Xuechao et al., 2019) (Figure 7A). They demonstrated that the temperature rose with increasing the concentration of Fe-soc-MOF@PPy from 31.25 μg/ml to 500 μg/ml, and the maximum temperature reached to 72.4°C, while it also has no obvious photothermal effects (Figure 7C). The results also show that the same amount of polypyrrole nanoparticles (approximately 75 μg/ml) and Fe-soc-MOF@PPy have similar photothermal effects. At the same time, the synthesized Fe-soc-MOF@PPy also showed a photothermal effect related to the laser power intensity (Figure 7D). The calculated photothermal conversion efficiency of Fe-soc-MOF@PPy aqueous solution is 13.9%. The results show that Fe-soc-MOF@PPy has stronger absorption than Fe-soc-MOF under the near-infrared spectrum of 808 nm, and the absorption intensity increases with the increase of Fe-soc-MOF@PPy concentration.

The body weight of the experimental group increased slightly during the treatment, and the biocompatibility of Fe-soc-MOF@PPy was good (Figure 7E). The tumor volume of the Fe-soc-MOF@PPy group was basically the same as that of the control group, but the tumor volume of the Fe-soc-MOF@PPy + NIR group was much smaller than that of the other groups. The results show that Fe-soc-MOF@PPy has good biocompatibility and can effectively inhibit tumor growth under laser irradiation. The combination of Fe nanoparticles and MOFs has many advantages: due to the low toxicity, good biocompatibility, and excellent photothermal effect, the as-synthesized Fe-soc-MOF@PPy nanocomposites could be used to inhibit and kill cancer cells efficiently under the 808 nm laser irradiation. This work extended and proves its feasibility of the LSS method to synthesize nano MOFs. The combination of MOFs with other functional materials shows prospective futures in construction of multifunctional theranostic agents for treatment of tumors and other applications.

### Polydopamine-Coated MOF for PTT

**FIGURE 8:**
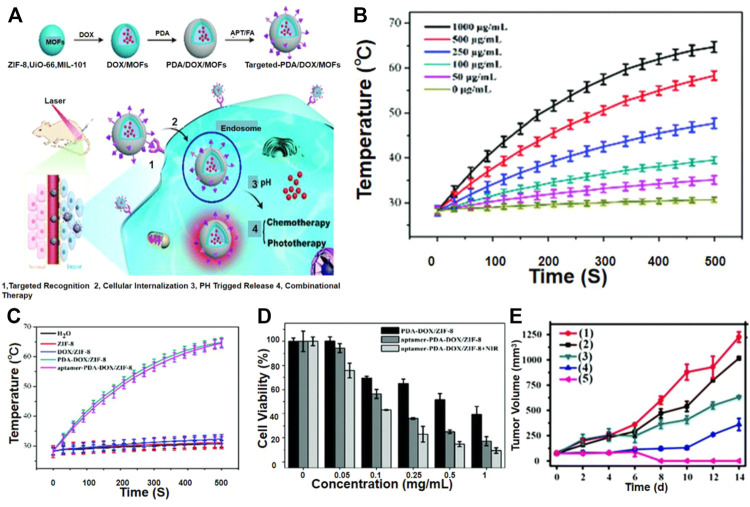
**(A)** Schematic MOFs as combinational therapy. **(B)** Heating curve of different concentrations of sgc-8-PDA-DOX/ZIF-8. **(C)** Heating curve of various components of PDA-DOX/ZIF-8. **(D)** Viability of HeLa cells with various concentrations of PDA–DOX/ZIF-8 and sgc-8–PDA–DOX/ZIF-8 in the presence or absence of 808 nm irradiation. **(E)** The change curve of tumor volume in mice after different treatments. Reproduced with permission (Feng et al., 2019). Copyright 2019, J. Mater. Chem. B.

Polydopamine (PDA), as a coating material, has been developed by combining MOFs for photothermal treatment (Liu et al., 2014c) Some groups reported that due to the unique and convenient coupling properties of PDA, the modified MOFs can be easily connected with functional molecules of interest. MOFs, such as ZIF-8, MIL-101, and UIO-66, once functionalized with PDA could couple with targeting molecules, such as aptamers and folic acid (FA), to achieve targeted drug delivery. Liu and his colleagues reported a multifunctional MOF material with a coated PDA for photothermal therapy (Figure 8A) (Feng et al., 2019).

Some researchers have proved that PDA@ZIF-8 can effectively convert 808 nm laser energy into ambient heat (Figure 8B), and the temperature rises rapidly after 500 s to reach a steady state. The temperature of the two materials has risen by about 36.5°C, while the temperature changes of the water, ZIF-8, and DOX@ZIF-8 systems are negligible (Figure 8C). These results confirmed that the light-to-heat conversion performance of ZIF-8 was attributable to the PDA coating. It has been reported that PDA can react with molecules containing nucleophilic groups, such as amines and thiols, through Michael addition or Schiff base reactions, and these molecules are easily combined through p-p interactions and hydrogen bonds (Park et al., 2014; Choi et al., 2015; Liu et al., 2016b). They proved that under laser irradiation, the viability of HeLa cells incubated with PDA-DOX@ZIF-8 was significantly reduced (Figure 8D).

Compared with the control group, they proved that under NIR irradiation, mice administered PDA@ZIF-8 had a higher inhibitory effect on tumor growth (Figure 8E). More importantly, PDA-DOX/ZIF-8 chemotherapy, photothermal therapy, and NIR light can cause tumor ablation. These results indicated that the combination of chemotherapy and phototherapy improved the anti-tumor activity and enhances the therapeutic effect. Other MOFs including ZIF-8, UIO-66, and MIL-101 could also be employed to prepare stimuli-response multifunctional hybrid material, which demonstrate the versatility of this strategy. This method enabled multiple treatments for targeted drug delivery and stimulus-response release and allowed the combination therapy to have good *in vitro* and *in vivo* anti-tumor activity. The combination of PDA and MOFs has many advantages: multifunctional MOF nanoparticles using PDA as a functional interface that affords facile conjugation with molecular units of interest as well as excellent photothermal transduction efficiency.

## Conclusion and Outlook

In this review, we have summarized a series of phototherapy agents based on MOF advanced materials. Compared with conventional PCAs, MOF-based PTA exhibited versatile advantages, including good biocompatibility, low toxicity, and enhanced photothermal conversion. The novel structure design combining metal core-shell nanomaterials with MOF shows great potential to regulate each component to achieve an enhanced photothermal conversion. There is a small limit for combined with photothermal nanomaterials with MOF; thus, the composition, size, and structure of metal particles, as well as the structure of the organic frame, could be independently constructed.

These PTAs based on MOF maintained their original photothermal properties, and the unique structure of MOF endowed photothermal reagents with great potential for various functionalization. Bulk MOFs and nanoscale MOFs (NMOFs) have exhibited many intriguing characteristics as drug carriers due to their low toxicity, exceptionally high surface areas, large pore sizes, and abundant functional groups on surface for drug loading *via* versatile interactions, such as vander Waals forces, π-π stacking, hydrogen bonds, electrostatic forces, and coordination bonds. To develop efficient delivery platforms, targeted delivery and stimuli-responsive release of various treatments will be effective for the treatment of cancer and other diseases. Besides, with post-synthesis modifications, MOF materials are ready to be endowed with various biomedical functionalities, such as bio-imaging and disease diagnosis. In order to create programmable MOFs for nanotherapeutic delivery and realize different functionalities, a universal method that can manipulate the surface of different MOFs is urgently needed.

On the other hand, post-synthetic modification by introducing well-designed functional groups into the organic linkers is a powerful strategy to improve the comprehensive performance of MOFs. MOF material is an excellent platform that could easily be modified with functional moieties to endow MOF hybrid material with specific properties. For example, once MOF being combined with functional groups (TCPP and UIO-66-AA), the obtained hybrid materials can produce singlet oxygen because of their inherent characteristics, which could be used as photosensitizers (PS) in PDT to kill nearby cancer cells by generating toxic singlet oxygen (SO). Accordingly, two categories of phototherapies (i.e., PDT and PTT) could work in a synergistic manner to kill cancer cells as the photothermal effect enhances the efficacy of PDT by relieving tumor hypoxia.
